# Study protocol: development and pilot testing of the Critical Care Pain Observation Tool for families (CPOT-Fam)

**DOI:** 10.1186/s40814-022-01102-3

**Published:** 2022-07-16

**Authors:** Anmol Shahid, Victoria S. Owen, Bonnie G. Sept, Shelly Longmore, Andrea Soo, Rebecca Brundin-Mather, Karla D. Krewulak, Stephana J. Moss, Kara M. Plotnikoff, Céline Gélinas, Kirsten M. Fiest, Henry T. Stelfox

**Affiliations:** 1 Department of Critical Care Medicine, Cumming School of Medicine, University of Calgary & Alberta Health Services, Calgary, Alberta Canada; 2 Faculty of Health, School of Health Administration, Dalhousie University, Halifax, Canada; 3 Centre for Nursing Research and Lady Davis Institute, Jewish General Hospital — CIUSSS West-Central-Montreal, Ingram School of Nursing, McGill University, Montreal, Canada; 4 Department of Psychiatry, Hotchkiss Brain Institute, Cumming School of Calgary, Calgary, Alberta Canada; 5 Department of Community Health Sciences, University of Calgary, Calgary, Alberta Canada; 6 O’Brien Institute for Public Health, Teaching, Research and Wellness Building, University of Calgary, Office 3E24, 3280 Hospital Drive NW, AB, T2N 4Z6 Calgary, Canada

**Keywords:** Pain assessment, Critical Care Pain Observation Tool, Family partnership, Intensive care unit pain, Tool development

## Abstract

**Background:**

Patients in the intensive care unit (ICU) often have limited ability to communicate making it more difficult to identify and effectively treat their pain. Family caregivers or close friends of critically ill patients may be able to identify signs of pain before the clinical care team and could potentially assist in routine pain assessments. This study will adapt the Critical Care Pain Observation Tool (CPOT) for use by family members to create the CPOT-Fam and compare family CPOT-Fam assessments with nurse-provided CPOT assessments for a given patient.

**Methods:**

This study will be executed in two phases:

1) Development of the CPOT-Fam — A working group of patient partners, ICU clinicians, and researchers will adapt the CPOT for use by family caregivers (creating the CPOT-Fam) and produce an accompanying educational module to deliver information on pain and how to use the tool. The CPOT-Fam will undergo preclinical testing with participants (i.e., members of the public and family caregivers of critically ill adults), who will complete the educational module and provide CPOT-Fam scores on sample cases. Feedback on the CPOT-Fam will be collected.

2) Pilot testing the CPOT — Fam family caregivers of critically ill adults will complete the educational module and provide information on the following: (1) demographics, (2) anxiety, (3) caregiving self-efficacy, and (4) satisfaction with care in the ICU. Family caregivers will then provide a proxy assessment of their critically ill loved one’s pain through the CPOT-Fam and also provide a subjective (i.e., questionnaire-based including open-ended responses) account of their loved one’s pain status. A comparison (i.e., agreement) will be made between family caregiver provided CPOT-Fam scores and ICU nurse-provided CPOT scores (collected from the provincial health information system), calculated independently and blinded to one another. Feasibility and acceptability of the CPOT-Fam will be determined.

**Discussion:**

The results of this work will produce a family caregiver CPOT (i.e., CPOT-Fam), determine feasibility and acceptability of the CPOT-Fam, and compare pain assessments conducted by family caregivers and ICU nurses. The results will inform whether a larger study to determine a role for family caregivers in ICU pain assessment using the CPOT-Fam is warranted.

**Supplementary Information:**

The online version contains supplementary material available at 10.1186/s40814-022-01102-3.

## Background

Many critically ill adults experience pain during their intensive care unit (ICU) stay [1–3]. Inadequately managed pain can arise from critically ill patients having limited ability to communicate, clinicians’ perceptions of analgesic therapy, and lack of patient education on analgesics [4, 5]. Inadequate pain management can lead to short- and long-term consequences for patients, their family caregivers (e.g., close family or friends), and health systems [6]. Deficiencies in pain management are associated with prolonged mechanical ventilation, longer hospital stays [7, 8], poor sleep and life quality [9], psychological distress [10, 11], and increased anxiety for patients and their families [12]. While ICU clinicians (e.g., physicians and nurses) aim to address concerns of pain thoroughly using standardized pain management guidelines [13–16], pain remains a major problem in ICUs and necessitates improved assessment and management [17].

Validated pain assessment tools such as the Behavioral Pain Scale (BPS), Behavioral Pain Scale Non-Intubated (BPS-NI), and the Critical Care Pain Observation Tool (CPOT) are commonly used by ICU clinicians to measure pain in patients with limited communication ability [18, 19]. Research in communicative patients has shown that family caregivers of critically ill adults are able to identify individualistic mannerisms that signal pain in the patient (e.g., facial expressions and redness, body movements) well due to their intimate previous knowledge of the patient [20]. Many studies have shown that adult-hospitalized patients’ self-described pain ratings align more closely with pain ratings by their family caregivers’ rather than those by nurses or physicians [21, 22]. This indicates an opportunity to partner with family caregivers in assessing pain in critically ill adults.

Family caregivers of critically ill patients are vital members of the ICU care team, often fulfilling roles ranging from providers of emotional support to surrogate decision-makers [23]. It is recommended that family members be included in patient care as this can improve trust and communication between family and healthcare providers and improve outcomes for patients [24, 25]. In ICU pain assessment, involvement of family caregivers can facilitate earlier pain recognition, reduce anxiety in family, and improve patient and family satisfaction with care [26]. The CPOT is a validated pain assessment tool for patients unable to self-report pain in ICUs [19] and may be suitable for use by family caregivers [27]. The CPOT is a routinely used four-item pain assessment tool with good specificity (78%) and sensitivity (86%), designed to be used by ICU clinicians on ventilated and non-ventilated ICU patients [18, 19, 28]. Each item on the CPOT can be scored from 0 to 2 which are added for a maximum score of 8, where a score of 3 or more indicates significant pain (i.e., moderate or severe) and warrants pain intervention [19]. CPOT scores have been shown to have a moderate correlation (0.59 and 0.71; *p* < .05) to self-reported pain intensity of ICU patients during painful procedures in the ICU (such as turning) [19]. Recent studies suggest that the CPOT may be amenable for use by family caregivers [27, 29]. However, some family caregivers have reported that identifying pain behaviors was stressful because they felt discomfort when assessing their loved one’s pain and did not find all elements of the tool relevant [27]. In this study, we intend to adapt the CPOT to family caregiver use (creating the CPOT-Fam) and pilot test the CPOT-Fam in adult ICU settings.

### Study aims

This study has two major aims:

1. Development of the CPOT-Fam: We aim to develop a family caregiver version of the CPOT (CPOT-Fam) accompanied by an educational module and conduct preclinical testing of the tool using sample cases of hypothetical critically ill adults.
2. Pilot test the CPOT-Fam: We aim to determine the feasibility and acceptability of the CPOT-Fam in adult ICU settings:

- Collect baseline measures of family caregivers’ anxiety, sense of self-efficacy to care for the patient, and satisfaction with patient care using validated tools.
- Determine agreement in CPOT scores between family caregivers and ICU nurses.
- Provide a narrative comparison between family caregivers’ subjective assessments of the critically ill patient’s pain (assessed using a short questionnaire) to family caregiver provided CPOT-Fam scores.

## Methods

**Fig. 1:**
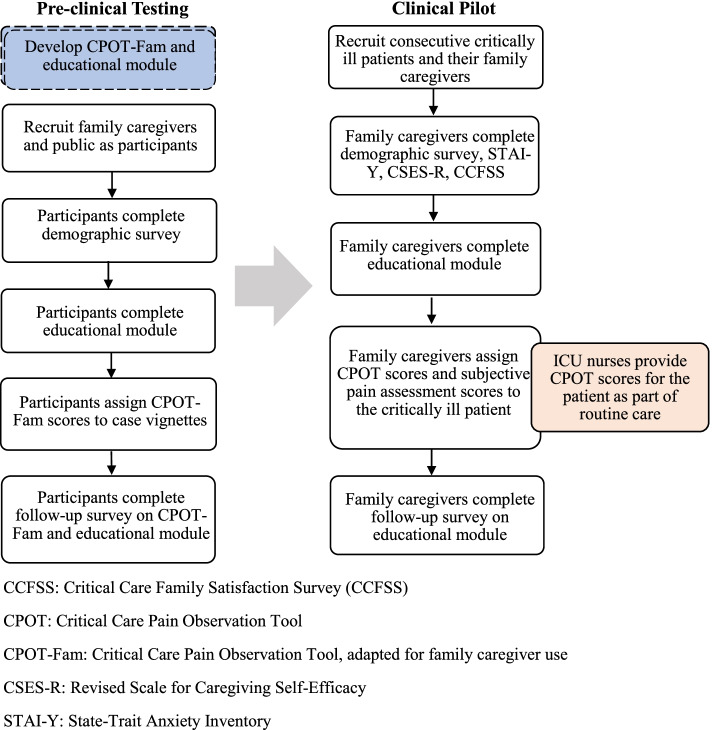
A flowchart of the study, reflecting the development of the CPOT-Fam and subsequent preclinical (left) and clinical pilot (right) testing phases

The two aims of this study will be executed sequentially. Methods for each aim are shown in Fig. 1 and described below.

### Development of the CPOT-Fam

#### Working group

To adapt the CPOT for use by families, we will convene a virtual working group with multiple stakeholders including patient partners (BS, SL), an ICU physician (HTS), an ICU nurse (VO), trainee researcher (AS), research staff (RBM, KK, KP, SM), and an experienced investigator (KF). The working group will meet monthly as follows: (1) determine the informational needs of family caregivers of critically ill adults regarding pain, (2) identify the steps required to adapt the CPOT to use by family caregivers, and (3) develop an accompanying pain education module to teach family caregivers to use the CPOT-Fam. After each working group meeting, minutes will be circulated by email to group members. The research lead (AS) will incorporate the feedback from group discussion to adapt the CPOT-Fam and build the educational module. Any redrafted contents will be circulated back to the group by email. This process will occur iteratively until a consensus is reached on the content of the CPOT-Fam and educational module.

#### Adapting the CPOT for family use

The CPOT is designed for use by ICU clinicians and contains complex medical terminology. To adapt the CPOT for use by family caregivers, the working group will discuss simplification of the CPOT to create the CPOT-Fam. The content areas and scoring of the CPOT-Fam will be identical to the CPOT, but the scoring descriptions will be modified to better suit (i.e., improve readability, clarity, and conciseness) family caregiver use.

#### Educational module content

The educational module will be developed as a multipart educational package which will include the following: (1) information on ICU pain, (2) information on how and when to use the CPOT-Fam, and (3) sample cases that family caregivers can score using the CPOT-Fam. A draft outline of the educational module containing further detail is presented in Table 1. The educational module will be written at a sixth-grade reading level and will be assessed by two research assistants (who are unfamiliar with the study) using the Patient Education Materials Assessment Tool for print materials (PEMAT-P) [30] to ensure that individuals with varying educational backgrounds and health literacy can understand the sample cases and utilize the information presented. The module will be considered appropriate for preclinical and clinical pilot testing if it meets criteria (≥ 70%) for understandability and actionability [31]. The educational module will be created to be administered in both web (i.e., narrated Microsoft PowerPoint video) and paper-based (i.e., printed Microsoft PowerPoint slides with captions) formats to suit participant preferences. The educational module will require 10 min to complete, regardless of the format.

**Table 1:** Proposed education module to accompany the CPOT-Fam and to be discussed and iteratively revised with a working group of stakeholders

A pain education module will be presented to consenting study participants. We will allow participants to access any section of the module repeatedly. The module will be broken down into two sections	
1) Introduction to ICU pain	• What is it? (What causes it, what kinds of pain to expect in the ICU) • Who is at increased risk? • How to distinguish ICU pain from expected discomfort • How to tell a member of the ICU care team that their patient may be experiencing pain
2) Assessing pain using the CPOT-Fam	• Basic overview of CPOT-Fam tool and how to use it • When it is appropriate to use it • Sample case

##### Sample cases

The sample cases will be based on four dimensions used to assess pain in the original CPOT: (1) facial expression, (2) body movements, (3) compliance with ventilator (if intubated) or vocalization (if not intubated), and (4) muscle tension [29]. Each sample case will represent a scoring option for dimensions 1–4, generating a unique combination adding up to a total CPOT score. For example, a sample case of a non-ventilated patient could reflect a total CPOT score of 3 and be comprised of individual dimensional scores such as the following: a score of 1 on *vocalization*, 2 on *facial expression*, 0 on *body movements*, and 0 on *muscle tension*. As such, there will be 81 unique combinations (4 dimensions across 9 scoring options = 81 combinations) of scores available for intubated patients and 81 for non-intubated patients for a total of 162 combinations. Of these, we anticipate some score combinations to be clinically unlikely (e.g., a score of 2 on *vocalization*, 0 on *facial expression*, 0 on *body movements*, and 0 on *muscle tension*) and suitable for removal, leaving approximately *n* = 120 sample cases. Clinically, unlikely scoring combinations will be identified through discussion with research team nurses and excluded. Family caregivers will be able to score pain for any given sample case by using the newly adapted CPOT-Fam. A sample case is attached in Supplementary item 1.

##### CPOT-Fam preclinical testing

After the CPOT-Fam and educational module are finalized, we will conduct preclinical testing with members of the general public and family caregivers of critically ill adults. Study participants will use the educational module and score the provided sample cases using the CPOT-Fam. This will be executed using a cross-sectional study design.

##### Study setting, participants, and recruitment

A group of participants for preclinical testing of the CPOT-Fam will be recruited from social media (e.g., Twitter channels), Bethecure.ca (a resource providing members of the general public information on participating in research and available opportunities), four adult medical-surgical ICUs, and one cardiovascular surgical ICU in Calgary, Canada. Participants will be eligible for inclusion in this study if they are 18 years of age or older, able to communicate in English (understand, read, speak), and able to provide informed consent. To minimize risk of COVID-19 exposure to study participants, study materials will be provided to participants of this phase digitally through email. All participants will provide verbal consent prior to participating.

##### Procedures

After recruitment, participants will be asked to complete the educational module before using the CPOT-Fam to score four sample cases. After this, participants will be asked to complete a short demographic questionnaire administered using Qualtrics (Provo, UT, USA) providing information such as their age, sex, highest level of education completed, language spoken at home, and their relationship with the patient in ICU (if applicable) (Supplementary item 2). Finally, participants will be asked to complete a short Qualtrics-based follow-up survey on the CPOT-Fam (Table 2). A research team member will facilitate participants’ completion of the educational module, sample cases, the demographic survey, and the follow-up survey by being present to answer questions by telephone or email. All data will be entered into a secure Research Electronic Data Capture (REDCap) database hosted at the University of Calgary, which provides an interface for validated data capture, audit trails for tracking data manipulation and export procedures, and seamless data downloads to common statistical software [32, 33]. For each sample case, participant CPOT-Fam scores will be compared with the reference standard CPOT score for a given sample case.

**Table 2:** Proposed follow-up survey to collect participants’ experience with the CPOT-Fam and accompanying educational module and their perceptions of pain in the ICU

A follow-up survey to solicit participants’ experiences with pain in the ICU and the pain education module (draft)	
**1) How comfortable do you feel in your ability to tell whether your family member is experiencing pain? (If applicable)** O Very comfortable O Moderately comfortable O Not comfortable or uncomfortable O Moderately uncomfortable O Very uncomfortable **2) If you identify pain in your family member, do you feel empowered to act on this information? (If applicable)** O. Yes O. No Why or why not? ____________________________________________________________________________________________________________________________________________________________________________________________________________________________________ **3) What would you do if you identified pain in your family member?** ____________________________________________________________________________________________________________________________________________________________________________________________________________________________________ **4) Please describe your experience with using the CPOT-Fam in a few words** ____________________________________________________________________________________________________________________________________________________________________________________________________________________________________ **5) Please describe your experience with the educational module (i.e., videos) in a few words** ____________________________________________________________________________________________________________________________________________________________________________________________________________________________________	

##### Sample size and power considerations

For preclinical testing of the CPOT-Fam, we aim to recruit participants according to an estimate of 0.85 for the intraclass correlation coefficient (ICC) between participant-assigned CPOT-Fam scores and reference scores (i.e., researcher-assigned scores). To estimate a 95% confidence interval with a width of 0.099 (± 0.050), we will require a total of *n* = 120 sets of scores from *n* = 30 subjects (*n* = 15 family caregivers of ICU patients, *n* = 15 members of the general public) based on each subject scoring 4 sample cases.

##### Statistical analysis

We will present the proportion of completion of the CPOT-Fam by family caregivers as a number and percentage. We will use the ICC to compare the participants’ CPOT-Fam scores with the reference CPOT-Fam scores. We will interpret the results according to the following accepted recommendations: an ICC ≤ 0.20 indicates slight agreement, 0.21–0.40 indicates fair agreement, 0.41–0.60 indicates moderate agreement, 0.61–0.80 indicates substantial agreement, and ≥ 0.81 indicates close to perfect agreement [34].

### Pilot testing the CPOT-Fam

After preclinical testing of the CPOT-Fam, the tool will be revised and pilot tested with family caregivers in adult ICU settings. These family caregivers will have a chance to learn to use the CPOT-Fam (with help from the educational module and sample cases) and practice using the CPOT-Fam to provide a proxy pain assessment when they visit their loved one in the ICU. Baseline measures for (1) family caregivers’ anxiety, (2) family caregivers’ sense of self-efficacy to care for the patient, and (3) family caregivers’ satisfaction with patient care will be collected. We will then compare family caregiver CPOT-Fam scores and nurse CPOT scores.

#### Study setting, participants, and recruitment

Consecutive critically ill adults and their family caregivers will be recruited from the four adult medical-surgical ICUs and one cardiovascular surgical ICU in Calgary, Canada. Patients will be eligible for inclusion in this study if they are 18 years of age or older, are being assessed for pain by nursing staff with the CPOT (i.e., are not able to communicate), and have a Richmond Agitation-Sedation Scale (RASS) [35] score of ≥ −4. Family caregivers of patients will be eligible for inclusion in this study if they are 18 years of age or older, are able to communicate in English (understand, read, speak), and are able to provide written informed consent.

ICU nurses will inform a research team member if a patient and family caregiver(s) meet the study criteria. A trained member of the research team will approach family caregivers of critically ill adults, provide them with information about the study, and, if the family caregiver agrees to participate, obtain written informed consent. If the family caregiver consents to participate in the study, the patient will be enrolled through surrogate consent as patients who meet the inclusion criteria will be noncommunicative.

#### Procedure and data collection

After recruitment, family caregivers will be asked to complete a demographic questionnaire providing information such as their age, sex, highest level of education completed, language spoken at home, and relationship with the patient (Supplementary item 2). Family caregivers will be asked to complete the State-Trait Anxiety Inventory (STAI-Y) [36, 37], the Revised Scale for Caregiving Self-Efficacy (CSES-R) [38], and the Critical Care Family Satisfaction Survey (CCFSS) [39, 40] to provide baseline measures of anxiety, self-efficacy, and satisfaction with care in the ICU [36–39, 41]. Following these assessments, a trained member of the research team will present the educational module to family caregivers via the web or in paper-based format, depending on the participant’s preference.

After receiving the educational module, the family caregiver will be asked to provide a proxy assessment of the patient’s pain by scoring a time-stamped CPOT-Fam during each of their visit(s) with the patient until the patient is discharged from ICU, up to a maximum of three visits. Family members will complete the CPOT-Fam only if the patients remain unable to communicate. At the same time, the family caregiver will be asked to provide a subjective assessment of the patient’s pain using a short questionnaire, which will include an open-ended question and a visual analog scale (shown in Table 3). Family caregivers will be asked to note whether a patient’s pain was assessed at rest or during a procedure to provide context for their score. All data will be entered into a secure REDCap database by the researcher.

**Table 3:**
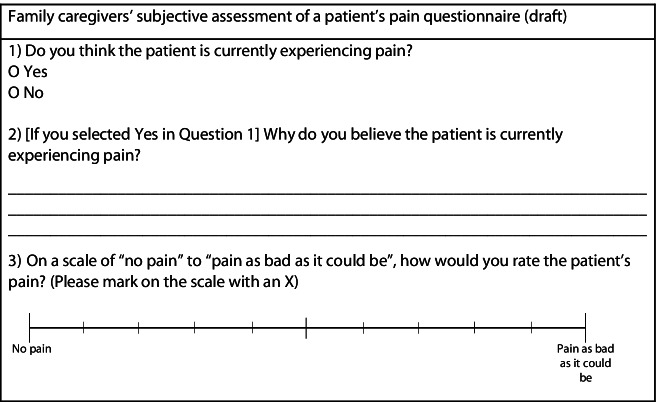
Proposed questionnaire to collect family caregivers’ subjective assessment of the critically ill patient’s pain [42]

Following the last CPOT-Fam assessment (i.e., after the patient is discharged from the ICU or after three assessments, whichever comes first), the family caregiver will be asked to complete a short follow-up survey (Table 2) to describe their experience with using the CPOT-Fam and their view of their role in assessing pain in their critically ill loved one. A research team member will be present to facilitate the completion of study tasks.

A trained research team member will coordinate with ICU nurses to provide their CPOT scores at the same time as the family caregivers provide their CPOT-Fam scores for a given patient on a given day. Assessments by family caregivers and ICU nurses will be conducted independently from one another. If this is not possible, the closest time-stamped CPOT score(s) by an ICU nurse will be pulled from eCritical, a population-based provincial critical care clinical information system which captures demographic, clinical, and outcomes data for all admitted ICU patients, including notes on sedation and pain [43]. Time-stamped CPOT scores by ICU nurses will be obtained, and any notes on sedation and pain will be reviewed to establish context for each CPOT score.

#### Sample size and power considerations

As this study is one of the first to adapt the CPOT to family caregivers and accompany it with an educational module [27], we aim to enroll a sample of 30 family caregivers based on projected timelines and enrollment rates in previous studies [44]. With a sample size of 30 participants, we will be able to estimate an acceptability measure of 50% (i.e., 15 out of 30) to within a 95% confidence interval (CI) of ± 18%.

#### Statistical analysis

We will calculate descriptive statistics (median (interquartile range), number (percentage)) for all study variables as appropriate. We will measure acceptability of the CPOT-Fam and educational intervention by calculating the proportion (with 95% CI) of eligible family caregivers approached who consented to participate in the study and feasibility by calculating the proportion (with 95% CI) of consented family caregivers who complete their participation in the study.

We will calculate intraclass correlation coefficients to measure agreement between CPOT-Fam scores by family caregivers and CPOT scores by ICU nurses. The data will be stratified based on time between CPOT-Fam scores by family caregivers and CPOT scores by nurses to determine whether agreement differs with the time that scores are obtained (i.e., closer together vs. further apart). A similar approach will be used to assess agreement between CPOT-Fam scores and subjective assessments by family caregivers.

## Discussion

### Deliverables and implications

The deliverable of this study will be a pain assessment tool suitable for family use (CPOT-Fam). This tool will aim to methodically engage family caregivers in assessing the pain of critically ill patients who cannot communicate. The CPOT-Fam and the results of this study could help advance the role of family partnership in ICU care. To our knowledge, this has not been done before. The results of this study will also inform whether a larger study to determine a role for family caregivers in ICU pain assessment is warranted, potentially informing practice implementation.

### Knowledge translation

Our team’s mandate is to build a strong role for family partnership in the care of critically ill adults. For the present study, we will utilize an integrated knowledge translation approach [45] and engage stakeholders who recognize that family caregiver participation in pain assessment could improve pain management in the ICU and consider this work a priority. These stakeholders have co-developed the research aims and procedures reflected in this study protocol. We will continue to engage stakeholders in all aspects of the study, including progress review, refinement of the methods, interpretation of data, and implementation of the lessons learned. Our team will disseminate the results of the present study into the broader national and international context using both traditional (e.g., manuscripts, presentations at conferences) and nontraditional (e.g., social media) dissemination strategies.

### Potential limitations

While the methodology of this study has been rigorously designed with multiple stakeholders, we recognize that this study has potential limitations. First, this study aims to involve family caregivers of critically ill adults, who are experiencing a stressful time. As such, the study may not be representative of family caregivers who decline to participate in the study or do not complete their participation in the study due to high anxiety or stress. To mitigate this limitation, we will make contact information for mental health support services available to interested family caregivers. Second, we will employ virtual participation to accommodate challenges to study enrolment posed by the COVID-19 pandemic. It is possible that different modes of participant engagement might influence how the CPOT-Fam is used. However, we believe this dual approach is necessary to allow a broad range of family caregivers to participate. Third, our study will compare family caregiver assessments of pain to those of ICU nurses, who may differ individually in their CPOT assessments. While this may be considered a limitation, this is the best reference standard available. Lastly, this study employs modest sample sizes (*n* = 30) for each of its phases; however, we believe this is adequate to provide us feedback on the development of the CPOT-Fam and determine whether further evaluation is warranted.

### Proposed timeline

We anticipate that adaptation of the CPOT for family use (CPOT-Fam), development of the educational module, and preclinical testing will take 4 months to complete. We estimate the recruitment, data collection, and data analysis of the pilot study will take an additional 4 to 6 months to complete.

## Supplementary Information

**Additional file 1: Supplementary item 1.** A sample case, reflective of sample cases that family caregivers will use to practice scoring on the CPOT-Fam. **Supplementary item 2.** Demographic questionnaire for participants of the study.

## Data Availability

Not applicable.
