# Supplementary material for: Study protocol: development and pilot testing of the Critical Care Pain Observation Tool for families (CPOT-Fam)
Source: Pilot Feasibility Stud. 2022 Jul 16;8:147. doi: 10.1186/s40814-022-01102-3 (PMC9287531; doi:10.1186/s40814-022-01102-3)
Supplement: Supplementary file 1 — Additional file 1: Supplementary item 1. A sample case, reflective of sample cases that family caregivers will use to practice scoring on the CPOT-Fam. Supplementary item 2. Demographic questionnaire for participants of the study. [file 40814_2022_1102_MOESM1_ESM.docx]

# SUPPLEMENTARY ITEMS

**Supplementary Item 1.** A sample case, reflective of sample cases that family caregivers will use to practice scoring on the CPOT-Fam.

Participant Study ID #:________________

Participant Initials: __________________

**Date: ____/____/_______ (dd/mm/yyyy)**

**Case 1**

**Patient: 45-year-old man**

**Reason for hospitalization: Life threatening infection**

**What you observed when you visited your loved one:**

You enter the room to visit your loved one. As you go closer to your loved one, you see that he is laying very still. He seems to be asleep and his face seems relaxed. He is breathing through a breathing tube but his breathing seems relaxed and easy.

**Supplementary Item 2.** Demographic questionnaire for participants of the study.

Demographic Questionnaire

Participant Study ID #:________________

Participant Initials: __________________

**Date: ____/____/_______ (dd/mm/yyyy)**

**Participant Role: Family Caregiver of patient (Family or Friend)**

(If known/applicable – the patient’s ICU dates)

ICU admission date: ____/____/_______ (dd/mm/yyyy)

ICU discharge date: ____/____/_______ (dd/mm/yyyy)

Duration in ICU _____________

**1) What is your age group?**

O <20 years

O 20-29 years

O 30-39 year

O 40-49 years

O 50-59 years

O 60-69 years

O 70-79 years

O ≥80 years

O Prefer not to answer

**2) What is your sex?**

O Male

O Female

O Prefer not to answer

**3) What is the highest level of education you have completed?**

O Less than a high school diploma

O High school diploma/CEGEP

O Vocational College

O Trade certification

O Some college or university

O College or university degree

O Higher education or professional degree

O Prefer not to answer

**4) What language do you speak most often at home?**

O English

O French

O Other (please specify): ___________

O Prefer not to answer

**5) What is your relationship to your loved one who is in the ICU? *(if applicable)***

O I am their spouse (including common-law)

O I am their parent

O I am their sibling

O I am their child

O I am their grandchild

O I am a close friend

O Other (please specify): ______________

O Prefer not to answer
